# Characterisation and pharmacological analysis of a crustacean G protein-coupled receptor: the red pigment-concentrating hormone receptor of *Daphnia pulex*

**DOI:** 10.1038/s41598-017-06805-9

**Published:** 2017-07-31

**Authors:** Heather G. Marco, Heleen Verlinden, Jozef Vanden Broeck, Gerd Gäde

**Affiliations:** 1 Department of Biological Sciences, University of Cape Town, Rondebosch, South Africa; 2 Molecular Developmental Physiology and Signal Transduction, KU Leuven, Naamsestraat 59, P.O. Box 02465, B-3000 Leuven, Belgium

## Abstract

This is the first pharmacological characterisation of a neuropeptide G protein-coupled receptor (GPCR) in a crustacean. We cloned the ORF of the red pigment-concentrating hormone from a German strain of *Daphnia pulex* (Dappu-RPCH), as well as that of the cognate receptor (Dappu-RPCHR). Dappu-RPCHR has the hallmarks of the rhodopsin superfamily of GPCRs, and is more similar to insect adipokinetic hormone (AKH) receptor sequences than to receptor sequences for AKH/corazonin-like peptide or corazonin. We provide experimental evidence that Dappu-RPCH specifically activates the receptor (EC_50_ value of 65 pM) in a mammalian cell-based bioluminescence assay. We further characterised the properties of the ligands for the Dappu-RPCHR by investigating the activities of a variety of naturally-occurring peptides (insect AKH and crustacean RPCH peptides). The insect AKHs had lower EC_50_ values than the crustacean RPCHs. In addition, we tested a series of Dappu-RPCH analogues, where one residue at a time is systematically replaced by an alanine to learn about the relative importance of the termini and side chains for activation. Mainly amino acids in positions 1 to 4 and 8 of Dappu-RPCH appear responsible for effective activation of Dappu-RPCHR. The substitution of Phe_4_ in Dappu-RPCH had the most damaging effect on its agonistic activity.

## Introduction

The common water flea *Daphnia pulex* (Class: Branchiopoda, Leydig 1860) is a planktonic filter-feeding crustacean that inhabits freshwater bodies. It forms an important part of the food chain and displays parthenogenetic reproduction under ideal environmental conditions^1, 2^. Daphnid crustaceans are model organisms in certain research fields, such as ecotoxicology, ecotoxigenomics and evolutionary ecology^3–6^. The entire genome of *D. pulex* is sequenced and represents the first crustacean genome available for data mining^7^, making it very interesting for comparative bioinformatic analyses, especially with a focus on peptide hormones^8, 9^. This class of hormones, specifically peptides originating from neuroendocrine centres, such as the X-organ – sinus gland complex, has been well-studied for decades in various infraorders of decapod crustaceans. Neuropeptide hormones play an important role in regulating all spheres of crustacean physiology (amongst others, development, metabolism, reproduction and growth). The physiological relevance of these neuropeptides could be examined with relative ease, but it was (and still is) difficult to make inroads into crustacean cell signalling where receptors for neuropeptide hormones are concerned^10^. Thus, the genome of *D. pulex* provides a window for comparative endocrinologists to look at crustacean peptide ligands and their receptors. The focus of the current paper is on one particular neuropeptide hormone signalling system in *D. pulex*, namely red pigment concentrating hormone (RPCH) and its cognate G protein-coupled receptor (GPCR).

RPCH is an octapeptide named according to its first known function in decapod crustaceans, *i.e*. causing translocation of red, yellow and brown pigment granules to a central spot in the cytoplasm of chromatophores, resulting in a pale-looking cell. If these pigment cells are located in the integument, RPCH results in overall blanching (whitening) of the decapod^11^. Panbo-RPCH, which was isolated from the prawn, *Pandalus borealis*, is the first invertebrate neuropeptide that was ever fully characterised and sequenced^12^. Subsequently, identical RPCHs were sequenced from a variety of decapod crustaceans belonging to different infraorders^13^. From available transcriptomes and expressed sequence tag (EST) data available since the last decade, it has been possible to deduce the RPCH sequence in other crustacean orders, and some were found to differ from Panbo-RPCH^14^.

RPCH is structurally similar to an insect neuropeptide, the adipokinetic hormone (AKH), which is produced in cerebral neurohaemal organs called the corpora cardiaca, and which has an effect on intermediate metabolism in insects^15^. Meanwhile, it is established that peptides identical to Panbo-RPCH are also synthesised in several insect species belonging to the orders Hemiptera, Plecoptera and Coleoptera, and, in a few of these species, these were demonstrated to mobilise lipids^14^. In a number of insect taxa gene duplication has taken place, with up to five AKH isoforms being produced in one species^16^, whereas crustaceans only produce one RPCH per species^14^. The RPCH/AKH peptide family has characteristic sequence features: a chain length of 8 to 10 amino acids, a blocked N-terminus (pGlu), a blocked C-terminus (carboxyamide), at least two aromatic amino acid residues (mostly Phe_4_ or Tyr_4_, and Trp_8_), the ninth residue (when present) is always Gly^13^. Several structure-activity relationship studies have been conducted in insects to ascertain the importance of specific amino acid residues of AKH for functional activity and such studies have also been extended to *in vitro* assays with the expressed AKHRs^14, 17–19^. Since all RPCH peptides in decapods identified to date have the same sequence, it is assumed that the decapod RPCHR is fairly conservative when it comes to binding RPCH/AKH ligands. Indeed, this was borne out in an *in vivo* study with the shrimp, *Palaemon pacificus*, where we demonstrated that Dappu-RPCH could not affect pigment migration in a decapod crustacean, whereas it caused measurable biological activity in the green stink bug, *Nezara viridula*, which has an AKH with the same sequence as Panbo-RPCH^11^.

Two other peptides in arthropods have structural similarity to the RPCH/AKH peptides: corazonin (Crz) and AKH/corazonin-related peptide (ACP)^20^. The function of Crz varies in different insect species, while the function of ACP is still unknown^20^. In certain insect species all three peptides occur^21^. A fourth peptide structurally related to RPCH/AKH peptides is gonadotropin-releasing hormone (GnRH)-like peptide. GnRH is a well-known master peptide in the reproductive cascade of vertebrates^22^. GnRH-like peptides (so named for their structural similarity) have been identified in several protostome groups^21, 23^. The giant freshwater prawn, *Macrobrachium rosenbergii*, possesses all four members of the so-called GnRH superfamily of peptides: RPCH, ACP, Crz and GnRH-like peptide^24, 25^.

AKHs exert their effects on cellular targets by binding to high affinity receptors, which are members of the superfamily of rhodopsin-like GPCRs and homologues of the vertebrate GnRH receptors^18, 19, 26–28^. Receptor sequences for RPCH in crustaceans may be deduced from genomic or transcriptomic information, based on sequence homology to other characterised receptors (notably the AKHR). A total of 85 GPCRs are predicted from *in silico* mining of the transcriptome of the spiny lobster, *Sagmariasus verreauxi*, one of which is considered an RPCH-like receptor^29^. Despite the paucity of information on crustacean RPCHR sequences, the RPCH signalling cascade has been investigated in several decapod crustacean species on a physiological and biochemical level^30^. From one such recent investigation with a G-protein antagonist, it was experimentally deduced that Panbo-RPCH lowers cAMP levels in ovarian chromatophores of the freshwater shrimp, *Macrobrachium olfersi* ^31^. In insects on the other hand, the AKHs seem to increase intracellular Ca^2+^ and cAMP levels^32^.

Crz and ACP receptor sequences are known from insects and not unsurprisingly, are structurally closely related to AKHRs, yet they are specific for their respective ligands^20, 26, 33, 34^.

The aim of the current study is to demonstrate that Dappu-RPCH and the GPCR deduced from the genome (Dappu-RPCHR) is a signalling pair. This has not been done before for any crustacean peptide ligand and cognate receptor. Here, we clone Dappu-RPCH and Dappu-RPCHR to affirm the structures predicted from the water flea genome, and characterise Dappu-RPCHR in an expression assay *in vitro*. This study shows unequivocally that RPCH (and not ACP or corazonin) is the ligand of this receptor. Ligand-receptor assays were also carried out to ascertain which ligand parameters are important for agonistic activity with this crustacean receptor.

## Results

### Cloning of Dappu-RPCH

**Figure 1:**
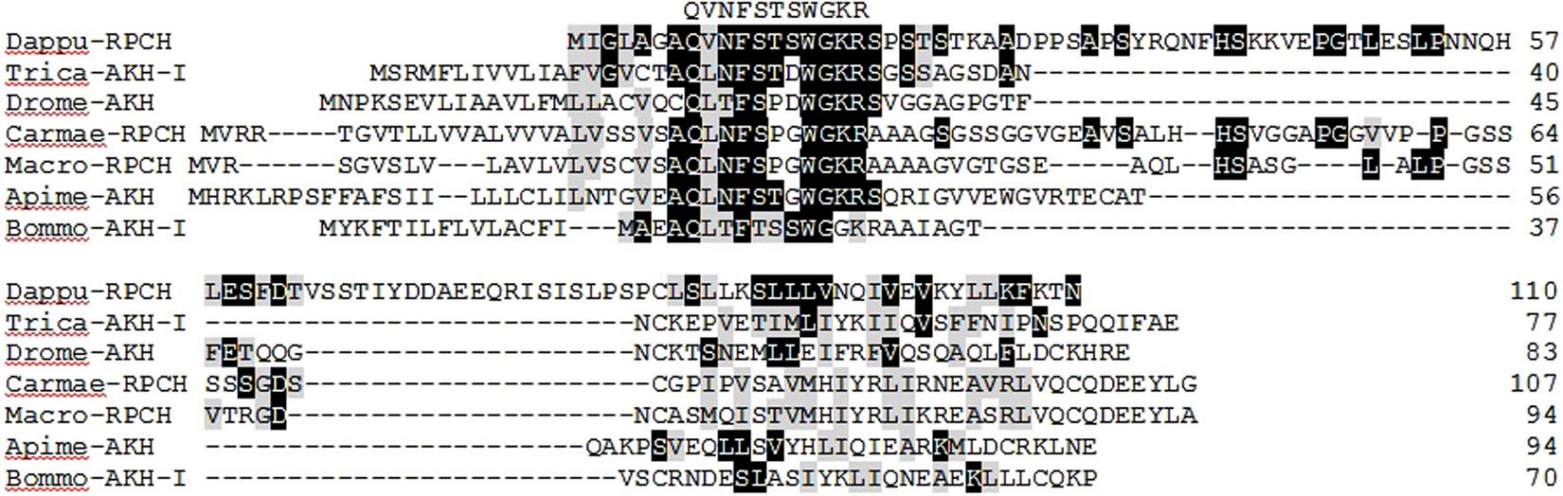
Amino acid sequence alignment of the precursors of Dappu-RPCH (GenBank acc. no. **ACJ05605**), Trica-AKH-I (GenBank acc. no. **ABN79648**), Drome-AKH (GenBank acc. no. **NP_523918**), Carmae-RPCH (GenBank acc. no. **AAB28133**), Macro-RPCH (GenBank acc. no. **ABV46765**), Apime-AKH (GenBank acc. no. **AEW68342**), Bommo-AKH-I (GenBank acc. no. **NP_001104825**). The amino acid position is indicated on the right. Identical residues between the Dappu-RPCH precursor and any of the other precursors are highlighted in black, and conservatively substituted residues in grey. Dashes indicate gaps that were introduced to maximise homologies. Putative transmembrane regions (TM1–TM7) are indicated by grey bars. Abbreviations used: Dappu, *Daphnia pulex*; RPCH, red pigment concentrating hormone; Trica, *Tribolium castaneum*; AKH, adipokinetic hormone; Drome, *Drosophila melanogaster*; Carmae, *Carcinus maenas*; Macro, *Macrobrachium rosenbergii*; Apime, *Apis mellifera*; Bommo, *Bombyx mori*.

The whole genome of the cladoceran crustacean *D. pulex* was sequenced and annotated by 2011^7, 9^. We searched the *D. pulex* genome database (wfleabase.org) with the genomic nucleotide scaffolds and the “Expressed Sequence Tag” (EST) selected in the “feature type” within the nBLAST program at wfleabase.org to identify an RPCH preprohormone. The predicted nucleotide sequence from the genome scaffold differed in size to the EST-derived sequence, with the former having a start codon further upstream from the EST start codon. A similar sequence with two putative start codons was also predicted from the genomic scaffold^8^, while a third prediction in NCBI database^9^ forecast a second methionine upstream from the EST start codon (Suppl. Fig. 1). Primers (Dpf and Dpr, Suppl. Table 1) were, thus, designed to first amplify the whole sequence of the EST-predicted RPCH preprohormone based on the genomic scaffolding sequence. This primer set amplified a 425 bp DNA product from the German *D. pulex* ecotype cDNA (whole animal) and the sequence (Suppl. Fig. 2) was reconfirmed by PCR-amplification with a high-fidelity Taq polymerase. The 333 bp open reading frame (ORF) of the amplified Dappu-RPCH encodes 110 amino acids: a short signal peptide (seven amino acid residues) and a long precursor-related peptide (83 amino acids) flank the Dappu-RPCH sequence with an amidation signal and dibasic cleavage site (Fig. 1; Suppl. Fig. 2). 5′ RACE PCRs were performed to amplify the predicted start codon(s) upstream of the start codon we had amplified, but all the attempts failed to amplify the extra amino acids. The 3′ sequence of the amplified ORF differs to all three predicted sequences, and three conservative amino acid substitutions are also noted in the translated precursor-related peptide (Suppl. Fig. 1). From the amplified ORF and from comparison with other members of the AKH/RPCH peptide family, it can be deduced that the mature Dappu-RPCH peptide has the following sequence: pQVNFSTSWamide (Fig. 1).

### Cloning of Dappu-RPCHR

**Figure 2:**
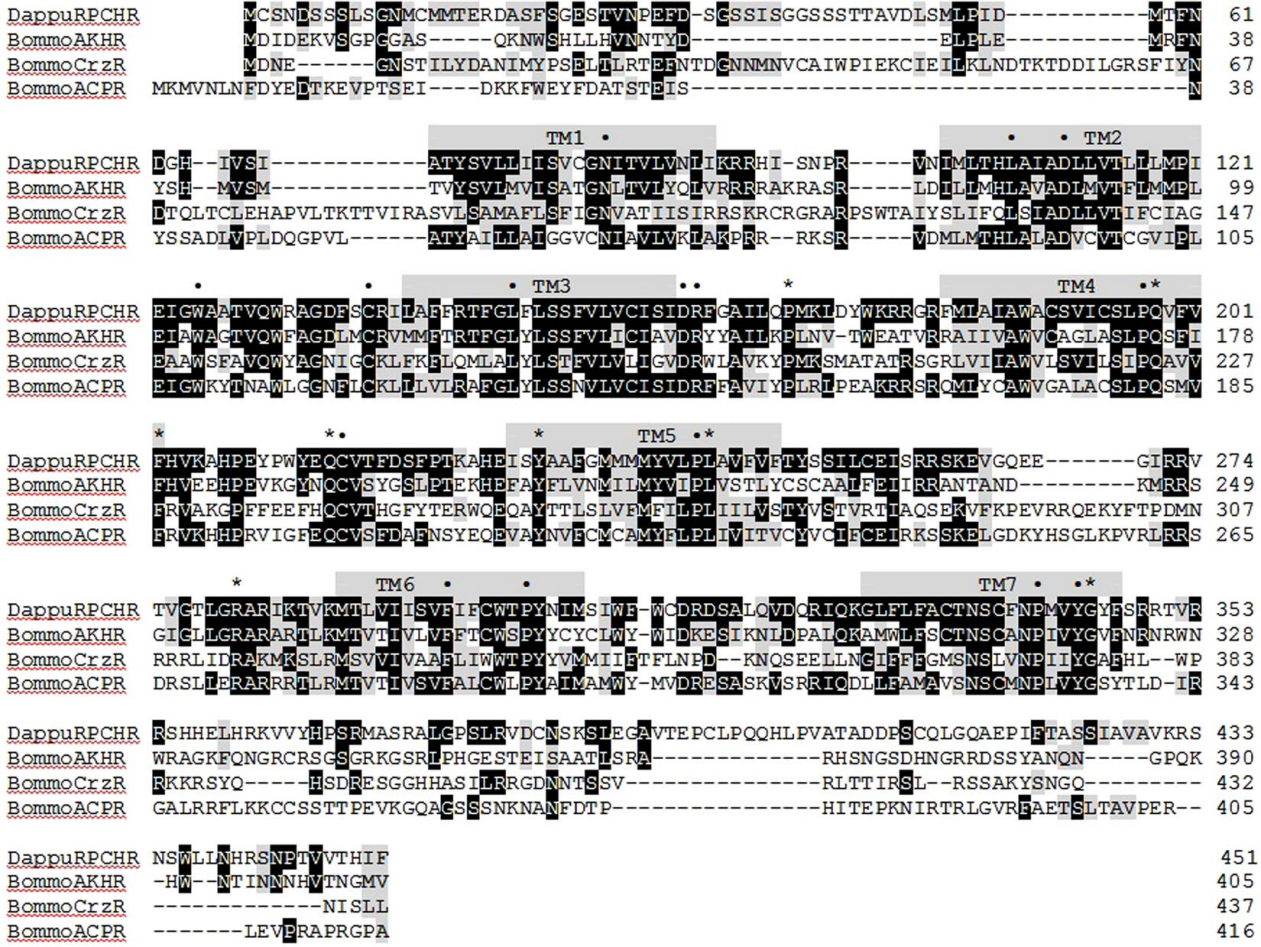
Amino acid sequence alignment of the Dappu-RPCHR (GenBank acc. no. **KY426816**), Bommo-AKHR (GenBank acc. no. **NP_001037049**), Bommo-CrzR (GenBank acc. no. **NP_001127719**) and Bommo-ACPR (GenBank acc. no. **ACT79362**). The amino acid position is indicated on the right. Identical residues between the Dappu-RPCHR and any of the other receptors are highlighted in black, and conservatively substituted residues in grey. Dashes indicate gaps that were introduced to maximise homologies. Putative transmembrane regions (TM1–TM7) are indicated by grey bars. Conserved amino acids for the rhodopsin-like GPCRs are indicated by a dot (•), whereas conserved amino acids for the GnRH receptor subfamily are indicated by asterisk. Abbreviations used: Dappu, *Daphnia pulex*; RPCHR, red pigment concentrating hormone receptor; Bommo, *Bombyx mori*; AKHR, adipokinetic hormone receptor; CrzR, corazonin receptor; ACPR, AKH/corazonin-related peptide receptor.

The *D. pulex* RPCH receptor was identified in the water flea genomic database (wfleabase.org) using an *in silico* search; five primers (Suppl. Table 1) were designed based on the genomic sequence, and the receptor cDNA was amplified via 5′ and 3′ RACE PCRs. The resulting RACE amplicons were sequenced and aligned to obtain a consensus sequence (Suppl. Fig. 3). RACE fragment sequences were verified in a nested PCR with a high-fidelity Taq polymerase to confirm the consensus sequence. The full length Dappu-RPCHR has an ORF of 1356 bp that encodes a receptor protein of 451 amino acid residues (Fig. 2; Suppl. Fig. 3; GenBank acc. no. **KY426816**). In addition, we amplified a further 572 bp that represents the 5′ untranslated region (UTR).

Seven membrane-spanning domains were predicted from the amplified Dappu-RPCHR sequence, with typical motifs that characterise the receptor as a rhodopsin-like GPCR (Fig. 2). The amplified translated Dappu-RPCHR protein sequence has high identity to *Daphnia* GPCR sequences that were obtained by conceptual translation from transcriptomes or genomes: 97% identity with a partial *D. pulex* “red pigment-concentrating hormone receptor” sequence (GenBank acc. no. **ACD75498**) and to the genome sequence (*D. pulex* **GNO_748024**; Suppl. Fig. 4), and 85% identity with a hypothetical GPCR 174 protein of *D. magna* (GenBank acc. no. **KZS09902**) and a “gonadotropin-releasing hormone II receptor” of *D. magna* (GenBank acc. no. **JAN73548**).

### Phylogenetic analyses

**Figure 3:**
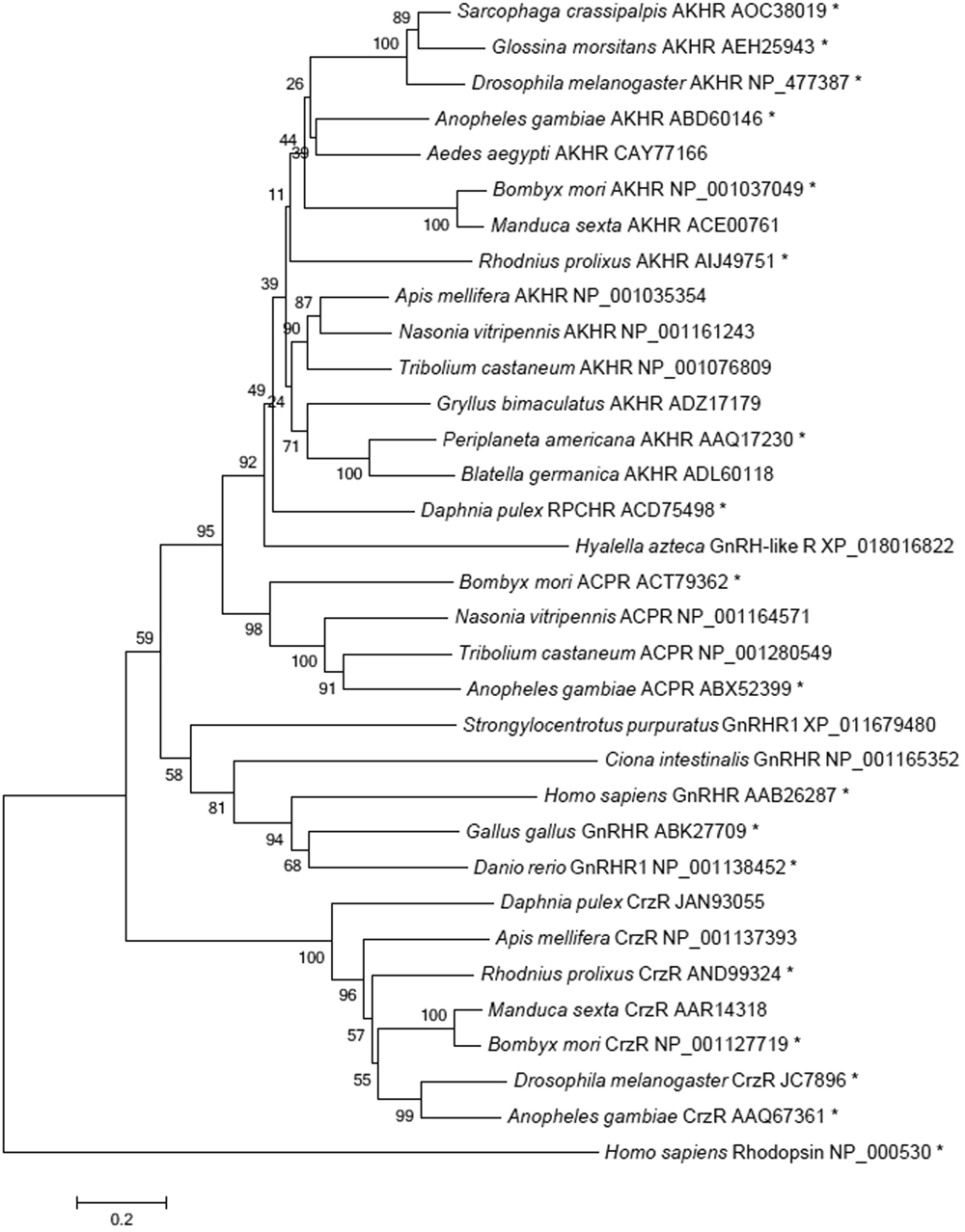
A neighbour-joining tree of GnRH receptor subfamily (i.e. GnRH/AKH/RPCH/ACP/Crz-type receptors). Phylogenetic and molecular evolutionary analyses were conducted by using MEGA vers. 6. Alignments were conducted with MUSCLE. Bootstrap-support values (1–100) are based on 1000 replicates using the Jones Taylor Thornton model and are indicated on the nodes. The human rhodopsin receptor was used as an outgroup to root the tree. Proteins marked with an asterisk were pharmacologically characterised. The scale bar allows conversion of branch length in the dendrogram to genetic distance between clades (0.2 = 20% genetic distance). Abbreviations used: AKHR, AKH receptor; RPCHR, RPCH receptor; ACPR, ACP receptor; CrzR, corazonin receptor; GnRHR, GnRH receptor.

Alignment of the precursor genes shows that only the neuropeptide RPCH/AKH sequence is very well conserved with a degree of conservation further downstream (Fig. 1). Consequently, phylogenetic trees result in very low Bootstrap values from which true relationships cannot be deduced^35^. The alignment of Dappu-RPCHR with the characterised receptors of *Bombyx mori* for AKH, ACP and Crz reveal the highest conservation in the sequence from the first to the last transmembrane region, with especially high conservation of the transmembrane regions themselves (Fig. 2). Several of the amino acid residues are marked in Fig. 2 by a dot (•), these belong to the hallmarks of the rhodopsin-like GPCR superfamily; whereas the ones marked with an asterisk appear to be characteristic for the GnRH receptor subfamily. Many of these subfamily-specific residues are located in or close by the extracellular loops of the GPCRs and are possible candidates for being ligand binding residues^35^. A phylogenetic tree of the receptor sequences indicates that RPCHR is more closely related to AKHR than to the other members of the GnRHR subfamily (Fig. 3 and Suppl. Fig. 5).

### Dappu-RPCHR is activated by peptides of the RPCH/AKH family

There are four peptide signalling systems in arthropods that have structurally related receptors and ligands, viz. RPCH/AKH, Crz, ACP and GnRH-like peptides; only the former two are present in *D. pulex* ^9^. We tested the specificity of the cloned Dappu-RPCHR in a cell-based bioluminescent assay with the following synthetic peptides (see Table 1 for sequence information): Dappu-RPCH, the *D. pulex* corazonin (Dappu-Crz) and an insect ACP (Locmi-ACP), as well as an insect AKH family peptide (Placa-HrTH, a decapeptide hypertrehalosaemic hormone from the cicada, *Platypleura capensis*) and other crustacean RPCHs (Panbo- and Argsi-RPCH).

**Table 1:** AKH/RPCH and related peptides tested on the putative *Daphnia pulex* RPCH receptor in *in vitro* bioluminescence assays.

Peptide name	Peptide sequence^1^	Native source	EC_50_ value (M)^2^
Dappu-RPCH	pQVNFSTSWamide	*Daphnia pulex*	6.45E-11
Argsi-RPCH	pQVNFST**K**Wamide	*Argulus siamensis*	2.13E-08
Anaim-AKH	pQVNFS**P**SWamide	*Anax imperator*	2.24E-10
Grybi-AKH	pQVNFST**G**Wamide	*Gryllus bimaculatus*	6.67E-11
Manto-CC	pQVNFS**PG**Wamide	*Order: Mantophasmatodea*	1.00E-10
Peram-CAH-I	pQVNFS**PN**Wamide	*Periplaneta americana*	3.93E-10
Libau-AKH	pQVNF**TP**SWamide	*Libellula auripennis*	1.79E-10
Placa-HrTH	pQVNFS**P**SWG**N**amide	*Platypleura capensis*	6.64E-10
Corpu-AKH	pQ**L**NFS**P**SWamide	*Corixa punctata*	2.12E-10
Schgr-AKH-II	pQ**L**NFST**G**Wamide	*Schistocerca gregaria*	2.37E-10
Panbo-RPCH	pQ**L**NFS**PG**Wamide	*Pandalus borealis*	7.93E-09
Nepci-AKH	pQ**L**NFS**SG**Wamide	*Nepa cinerea*	2.72E-10
Locmi-ACP	pQVTFSRDWSPamide	*Locusta migratoria*	n.a.
Dappu-Crz	pQTFQYSRGWTNamide	*Daphnia pulex*	n.a.

Abbreviations used: RPCH, red pigment concentrating hormone; AKH, adipokinetic hormone; CC, *corpora cardiaca*; CAH, cardioacceleratory hormone; HrTH, hypertrehalosaemic hormone; ACP, adipokinetic/corazonin-related peptide and Crz, corazonin; n.a., not applicable.

^1^Amino acid residues in bold text indicate a substitution in RPCH/AKH sequences relative to Dappu-RPCH.

^2^EC_50_ values were calculated from dose-response curves in the current study.

**Figure 4:**
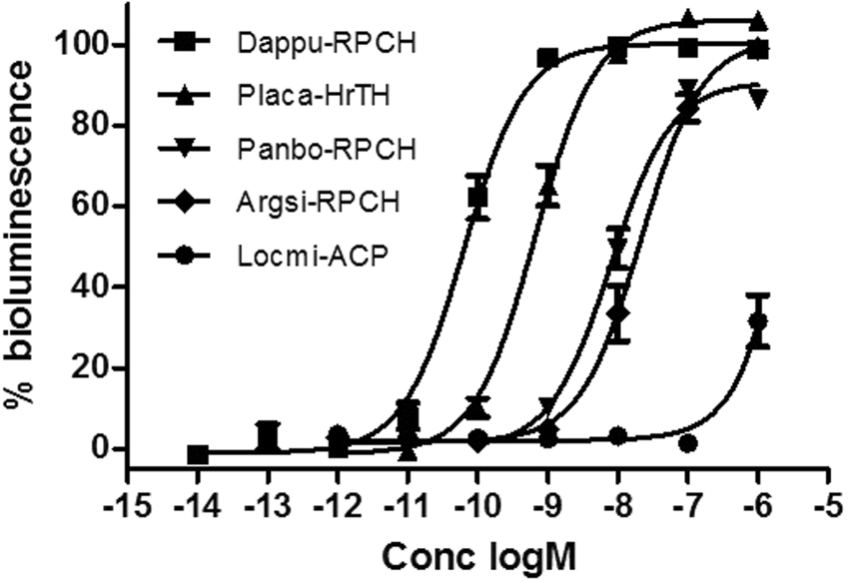
Dose-response curves of Dappu-RPCH and other structurally related peptides tested on Dappu-RPCHR expressing cells in an *in vitro* bioluminescent assay. Data shown as Mean ± SD, each experiment was performed in triplicate, and % bioluminescence is a proxy for receptor activation. Table 1 shows peptide structure and EC_50_ values.

Dappu-Crz did not activate the receptor, while Locmi-ACP reached only 30% of the maximum activation at a high peptide concentration of 10 µM (Fig. 4). Dappu-RPCH, on the other hand, clearly bound and activated the receptor with an EC_50_ value of 65 pM (Fig. 4, Table 1). These results indicate that the octapeptide Dappu-RPCH, is the endogenous ligand for the cloned GPCR, Dappu-RPCHR.

Other crustacean RPCHs were less effective at activating the water flea receptor than the endogenous ligand, Dappu-RPCH. Panbo-RPCH (the RPCH in decapod crustaceans) with three amino substitutions, Leu_2_, Pro_6_ and Gly_7_, had an EC_50_ value of 7.93 nM, while Argsi-RPCH (the peptide from the brachyuran carp louse), with only one amino acid substitution (Lys_7_), showed a slightly lower efficacy with an EC_50_ value of 21.3 nM (Fig. 4, Table 1).

### Receptor activation by naturally-occurring AKH ligands

**Figure 5:**
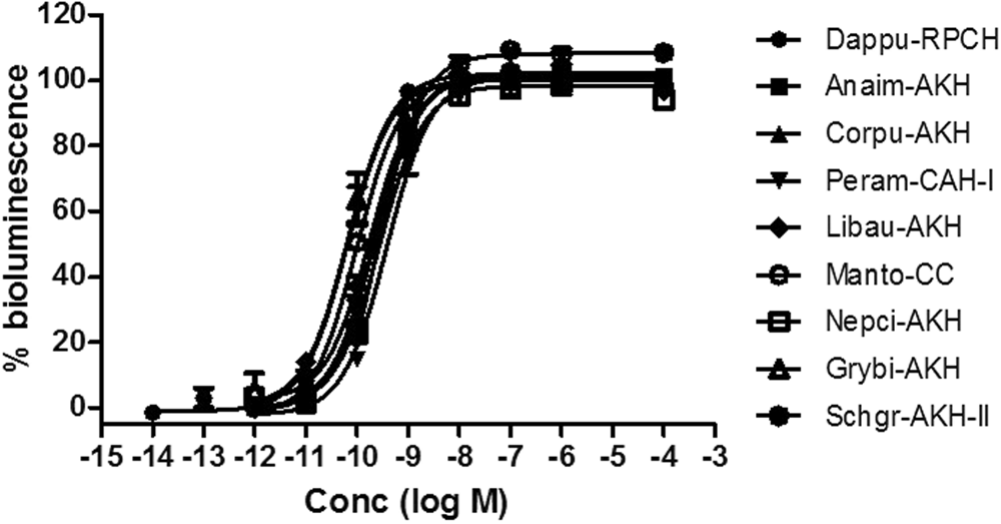
Dose-response curves of Dappu-RPCH and structurally related insect AKH peptides tested on Dappu-RPCHR expressing cells in an *in vitro* bioluminescent assay. Data shown as Mean ± SD, each experiment was performed in triplicate, and % bioluminescence is a proxy for receptor activation. Table 1 shows peptide structure and EC_50_ values.

We selected a number of AKH family octapeptides that are present in insect species and have one to three amino acid substitutions relative to Dappu-RPCH, particularly substitutions at positions 2, 6 and 7 (see Table 1 for structures) to try to understand the loss of potency demonstrated by the two crustacean RPCHs above. Figure 5 shows that the selected insect AKH peptides all activate the Dappu-RPCHR in a typical dose-related manner and are fairly potent in activating the receptor of the water flea, despite their amino acid substitutions: single amino acid substitutions, such as in Grybi-AKH (Ser_7_ to Gly_7_) of the cricket *Gryllus bimaculatus*, or Anaim-AKH (Thr_6_ to Pro_6_) of the dragonfly *Anax imperator*, had no effect or only a slight effect on activating the receptor (EC_50_ of 67 and 224 pM, respectively; Table 1). When a substitution was introduced in addition to Pro_6_, such as Leu_2_ (Corpu-AKH of the water boatman *Corixa punctata*), Asn_7_ (Peram-CAH-I of the cockroach *Periplaneta americana*) or Gly_7_ (Manto-CC of the heelwalker *Order: Mantophasmatodea*), the EC_50_ values were more or less the same as for Anaim-AKH (Table 1). Similarly, limited changes in efficacy (*i.e*. about 4 x less than Dappu-RPCH) were noted with Schgr-AKH-II (Leu_2_ and Gly_7_ of the desert locust *Schistocerca gregaria*) and Nepci-AKH (Leu_2_, Ser_6_ and Gly_7_ of the water scorpion *Nepa cinerea*) (Table 1, Fig. 5).

We also tested the effect of an extended peptide chain length on activating Dappu-RPCHR by applying the decapeptide hypertrehalosaemic hormone (Placa-HrTH) to the receptor-expressing cells. Placa-HrTH is near-identical in sequence to Dappu-RPCH – it has the same sequence as Anaim-AKH but is extended C-terminally with G_9_N_10_amide (Table 1). The results indicate that the extended peptide chain length has an effect on activating Dappu-RPCHR. Figure 4 shows that this extension reduces the agonistic potential *ca*. 10-fold, as an EC_50_ value of 0.66 nM was measured (Table 1).

### Structure-activity analysis with modified Dappu-RPCH ligands

**Figure 6:**
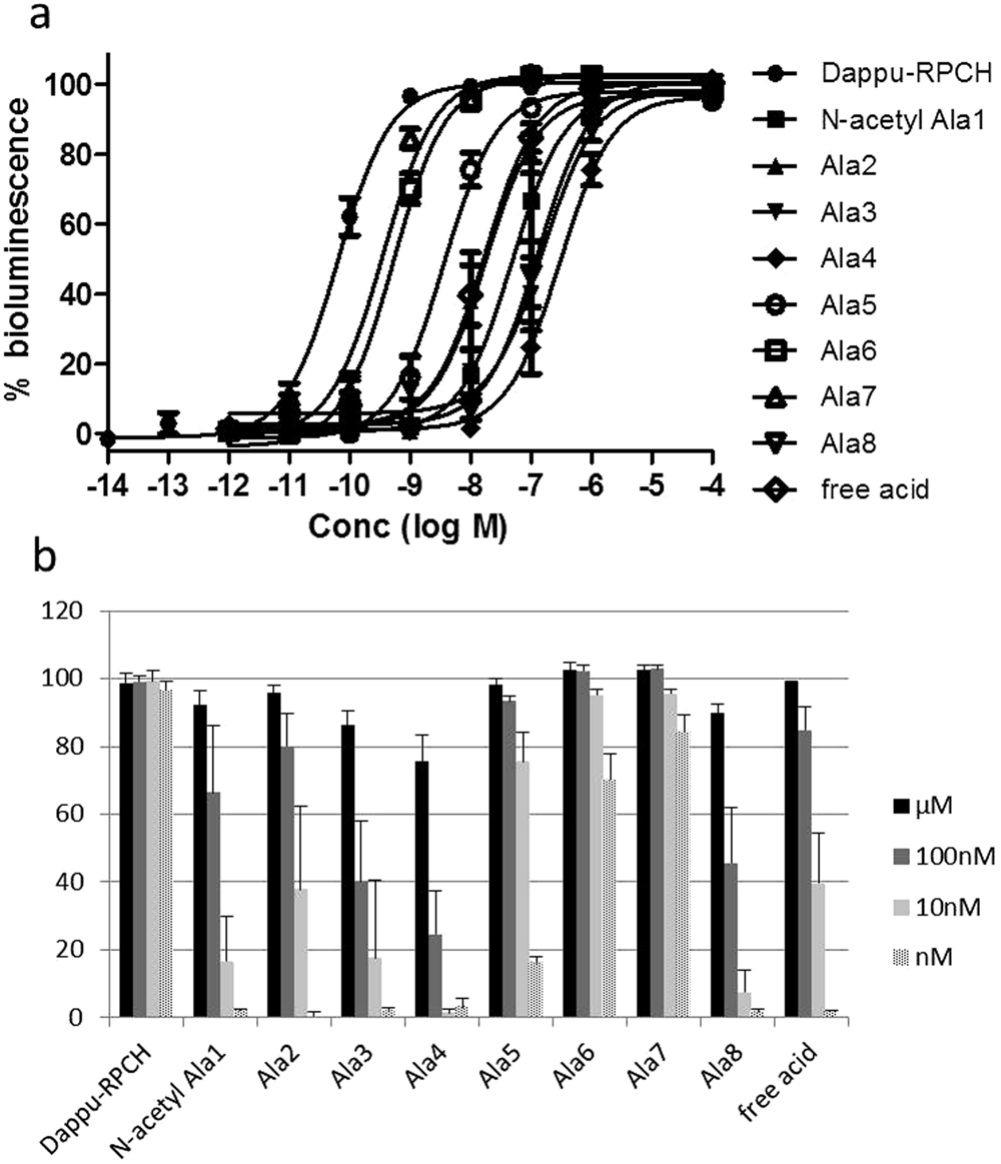
Structure-activity relationship of Dappu-RPCH and analogues thereof tested on Dappu-RPCHR expressing cells in an *in vitro* bioluminescent assay. (**a**) Full structure-activity curves; data shown as Mean ± SD, each experiment was performed in triplicate, and % bioluminescence is a proxy for receptor activation. (**b**) Receptor activation data shown as a histogram in the micro- to nanomolar range for each Dappu-RPCH analogue. Table 2 shows peptide structure and EC_50_ values.

To determine the relative importance of the eight amino acids making up the endogenous ligand Dappu-RPCH with respect to receptor activation, a series of synthetic Dappu-RPCH analogues was designed; each analogue had one amino acid substituted with a simple alanine residue (Table 2), and was tested in the cellular bioluminescence assay to activate the expressed Dappu-RPCHR. In this way we can explore the relative importance of each side chain and the blocked termini for activating the Dappu-RPCH receptor. A marked reduction of the EC_50_ values resulted when the N-terminal pGlu residue was replaced with a blocked Ala (N-acetyl Ala), or when the amidated C-terminus was replaced with a carboxyl group (free acid) (Table 2), thus demonstrating a 750-fold and 270-fold, respectively, lower affinity of these peptides for Dappu-RPCHR (Fig. 6A). Replacing Val_2_ with Ala_2_ had a similar effect as the unblocked C-terminal Dappu-RPCH analogue (Table 2; Fig. 6B). Changes at positions 3 (Asn to Ala), 4 (Phe to Ala) and 8 (Trp to Ala) all resulted in a very pronounced decrease in receptor activation (Fig. 6B), with EC_50_ values in the low micromolar range (Table 2). Substitutions of Ser_5_ with Ala, and especially Thr_6_ and Ser_7_ with Ala, were particularly well-tolerated by the receptor, and had only a relatively small effect on EC_50_ values (Table 2, Fig. 6B).

**Table 2:** Analogues of Dappu-RPCH tested on the *Daphnia pulex* RPCH receptor in *in vitro* bioluminescence assays and the resulting EC_50_ values.

Peptide name	Peptide sequence	EC_50_ value (M)
Dappu-RPCH	pQVNFSTSWamide	6.45E-11
[N-Ac-Ala^1^]Dappu-RPCH	**[NAc-Ala]**-VNFSTSWamide	4.87E-08
[Ala^2^]Dappu-RPCH	pQ**A**NFSTSWamide	1.77E-08
[Ala^3^]Dappu-RPCH	pQV**A**FSTSWamide	1.56E-07
[Ala^4^]Dappu-RPCH	pQVN**A**STSWamide	2.99E-07
[Ala^5^]Dappu-RPCH	pQVNF**A**TSWamide	3.61E-09
[Ala^6^]Dappu-RPCH	pQVNFS**A**SWamide	5.32E-10
[Ala^7^]Dappu-RPCH	pQVNFST**A**Wamide	3.55E-10
[Ala^8^]Dappu-RPCH	pQVNFSTS**A**amide	1.31E-07
[Trp-OH]Dappu-RPCH	pQVNFSTS**W**-**OH**	1.74E-08

## Discussion

Several sequence predictions have been made for the preprohormone sequence of the red pigment-concentrating hormone in *D. pulex* (Dappu-RPCH) and it’s GPCR (Dappu-RPCHR). Here we report on sequences deduced from PCR amplification with several small differences as compared to the predicted ones, but with demonstrated functional activity.

The very short signal peptide of Dappu-RPCH amplified in the current study is unusual as AKH/RPCH signal peptides are around 20 amino acids long (Fig. 1). The sequence predicted from the genomic scaffold has a longer signal peptide but 5′ RACEs of the current study were not successful in amplifying the predicted 5′ end. This may possibly indicate a fault in the genomic data, e.g. misassembly of the read sequences, or perhaps poor quality of the RNA extracted from *D. pulex* specimens in the current study, although RNA was extracted from several batches of animals and the much longer receptor cDNA was also cloned from it. It is interesting, too, that a previous attempt to amplify the predicted Dappu-RPCH preprohormone by RT-PCR was unsuccessful^9^, and that an earlier *in silico* search of *D. pulex* ESTs reported that Dappu-RPCH could not be found^36^. Nonetheless, the RPCH preprohormone of *D. pulex* amplified in the current study is constructed in the same way as for other AKH/RPCHs, viz. a signal peptide, Dappu-RPCH, amidation and dibasic cleavage site, and finally a precursor-related peptide. The translated, mature Dappu-RPCH peptide has the following sequence: pQVNFSTSWamide. The presence of such a peptide in a United Kingdom *D. pulex* clone (strain Livpu01) was deduced from mass spectrometry as [M + H]^+^ = 950.44, although the mass ion was not fragmented to confirm the amino acid sequence and peptide identity^9^.

We also cloned a putative Dappu-RPCHR and as with Dappu-RPCH, there are several small sequence differences (Suppl. Fig. 4). However, since the cloned receptor is functional *in vitro*, we believe the amplified receptor is complete and that there may be a mistake in the assembled shotgun genomic sequence, or that the observed differences may be attributed to maternal clonal variation from which the starting material was derived. The amplified receptor shows the hallmarks of a rhodopsin-like GPCR and, when we look at an alignment of Dappu-RPCHR with three pharmacologically characterised receptors of the silk moth *B. mori*, viz. Bommo-AKHR, -CrzR and –ACPR (Fig. 2), the greatest resemblance is to Bommo-AKHR (46% identical and 66% similar residues), followed by Bommo-ACPR (42% identical and 64% similar residues). Bommo-CrzR shows least resemblance with 32% identical and 53% similar residues (Fig. 2). Bommo-AKHR and Dappu-RPCHR sequences were also aligned with the incomplete, predicted, putative RPCHR sequence from the spiny lobster *S. verreauxi* ^29^, as this is the only other crustacean putative RPCHR structure available for comparison (see Suppl. Fig. 5). From the overlap between all three sequences, it seems that the banchiopod crustacean receptor shares more identical amino acid residues with the insect AKHR than with the decapod crustacean’s putative RPCHR. Until we have more sequence information from characterised crustacean RPCHRs, this can only be a tentative interpretation.

In the phylogenetic tree of members of the GnRH receptor subfamily it is evident that Dappu-RPCHR falls within the cluster of AKH receptors, and that it is more closely related to ACP receptors than to Crz receptors (Fig. 3). To confirm that the cloned Dappu-RPCHR is only activated by RPCH/AKH and not the other members of the GnRH subfamily of peptides, a selection of peptides were presented to the expressed receptor in a cellular assay: it should be noted that ACP and a GnRH-like peptide have not been identified in *D. pulex* to date; nevertheless, we included an ACP from the migratory locust in this receptor validation experiment. The specificity of the receptor for RPCH/AKH was very clear: whereas endogenous Dappu-Crz could not activate the Dappu-RPCHR at all and Locmi-ACP was only slightly active at concentrations of 10 µM, full activity with Dappu-RPCH was achieved by 0.1 nM. This then characterises the cloned receptor as the first RPCH receptor that is subjected to detailed structure-activity investigations. Some insect receptors of the GnRH subfamily have been investigated extensively, such as from *B. mori* ^34^, *Anopheles gambiae* ^20, 33^ and *Drosophila melanogaster* ^26^, and have also been shown to be specific for either AKH, ACP or Crz. Apparently this ligand-specificity is shared in the crustacean *Daphnia* in as much as that its RPCH receptor is specific for only RPCH/AKHs, and Dappu-RPCH and its receptor form a tight and specific signalling system (Figs 4 and 5). Our data thus support the notion of receptor-ligand co-evolution resulting in independent signalling systems^23, 35^.

We have shown quite clearly for the Dappu-RPCH receptor in the present study which amino acid residues of the ligand are important for agonistic activity and also how important the blocked termini are. The latter are also considered important for prolonging the half-life of the AKH/RPCH peptides while circulating in the haemolymph of the insect or crustacean. In our *in vitro* studies, however, the enzymatic component and extended time to reach the receptor are no longer factors for consideration, yet Dappu-RPCH with the unblocked C-terminus shows severely reduced activity (270-fold) and the (N-acetyl Ala) substitution for pGlu_1_ results in an even more severely reduced EC_50_. Since there is limited opportunity for enzymatic degradation of the peptide, it is very likely that the observed reduction in agonistic potency is due to steric hindrance at the receptor, or a conformational change brought on by the altered terminus, or the negative charge introduced by the unblocked C-terminus, which may have resulted in a reduced affinity to the ligand-binding pocket of the receptor. Other important amino acid residues for activation of Dappu-RPCHR are similar to what has been identified in several insect studies^17–19^, viz. the aromatic amino acids: phenylalanine at position four and the tryptophan at position eight are crucial for binding to/activation of the receptor. Ala substitutions at positions two and five hampered receptor activation but not to the same severe extent as the Ala substitution at position three. These results are very similar to what was observed with modified *D. melanogaster* AKH and structure-activity relationship studies with the cognate AKHR^18^, and may be explained by the impeded secondary structure of AKH/RPCH that the substitutions bring about.

In contrast, Ala substitutions at positions five, six and seven of Dappu-RPCH were particularly well-tolerated by the receptor, and had only a small effect on EC_50_ values (Table 2; Fig. 6), hence, these residues are less important for binding the receptor. This is reflected to an extent by the activation of the Dappu-RPCH receptor by a variety of AKHs from insects and crustaceans (Table 1; Fig. 5). Amino acid substitutions at position 7 were tested: Gly_7_ and Asn_7_ for Ser_7_ had relatively little consequence, whereas Lys in position 7 (in the form of the crustacean peptide Argsi-RPCH) did not perform well in the receptor assay, and this is most likely due to the fact that lysine is a positively charged, bulky amino acid, which will severely disrupt the secondary structure of the peptide, as well as its intermolecular interactions with the receptor. The substitution of Thr in position 6 with Pro did not cause much of a disturbance in peptide interaction with Dappu-RPCHR, but in combination with Leu_2_ and Gly_7_ (Panbo-RPCH) a noticeable impact was made, which contrasted strongly with Manto-CC (Val_2_, Pro_6_, Gly_7_) (Table 1), and indicates the vulnerability of certain substitutions at position 2 of the AKH/RPCH peptides.

Clearly, the Dappu-RPCHR has “preferred” ligands as demonstrated in the current study, and Panbo-RPCH is not one of those (Table 1). In fact, the ligands that perform best in the current cell-based assay with Dappu-RPCHR are the ones that performed worst in a functional *in vivo* assay with the shrimp *Palaemon pacificus* that is responsive to Panbo-RPCH^11^. The current results are, thus, confirmation that the RPCH receptor in decapod crustaceans requires different sites on the ligand for binding and activation than do the AKH receptors in insects and the RPCH receptor in the branchiopod crustacean. It is thus, very interesting to see that *Daphnia* appears to be more closely related to the insects than to the higher crustacean order, the decapods^29, 35^.

## Materials and Methods

### Animals

Laboratory-reared adult *D. pulex* were kindly supplied by Dr Bettina Zeis (Zoology Department, University of Münster, Germany). This ecotype had originally been collected from streams and river beds in Gräfenhain, Sachsen, Germany. Whole animals were immersed in RNAlater® (Applied Bioscience – Ambion®) until use for the initial amplification of the RPCH precursor and its receptor.

### Cloning and sequencing of *D. pulex* RPCH and the RPCH receptor

The predicted RPCH preprohormone of *D. pulex* (Dappu-RPCH) and the predicted RPCH receptor (Dappu-RPCHR) were identified from nBLAST searches of the waterflea genomic database available at http://wfleabase.org. Primers (Suppl. Table 1) were designed based on these *in silico* identifications.

Total RNA was extracted from whole animals using Total RNA Isolation Reagent® (TRIR) (ABgene). Animals were briefly homogenized in 1 ml TRIR reagent in Eppendorf tubes with a plastic pestle. Total RNA was resuspended in 0.1% DEPC (diethylpyrocarbonate) treated water and stored at −80 °C. The resuspended RNA was treated with DNase (Fermentas), and thereafter, cDNA was synthesised using the SuperScript™ III First Strand Synthesis System for RT-PCR (Invitrogen).

### Amplification of *D. pulex* RPCH (Dappu-RPCH)

For the amplification of Dappu-RPCH the primer set Dpf and Dpr was used. Amplification was achieved with MyTaq™ DNA Polymerase (Bioline) and the following PCR conditions: initial denaturation at 95 °C for 2 min followed by 35 cycles of 95 °C for 15 s, 52 °C for 30 s (annealing) and 72 °C for 10 s (elongation), and a final elongation step of 72 °C for 10 min. PCR products were separated in a 2% agarose gel, excised and the DNA was extracted with the Wizard® SV Gel and PCR Clean-Up System (Promega Corporation). DNA amplicons were sub-cloned into the pGEM-T Easy vector system (Promega Corporation). DH_5α_ *Escherichia coli* cells were transformed with the recombinant vector according to instructions in the pGEM-T Easy vector manual. Plasmid DNA extraction was carried out with the BioSpin plasmid DNA extraction kit (Bioflux, Bioer Technology Co.) and sent for commercial sequencing using M13F and/or M13R primers (Macrogen, Korea). Sequence data were analyzed using the DNAMAN (Lynnon, Quebec, Canada) and BioEdit bioinformatic tools^37^. Homology searches were conducted using the Blast® programs, namely blastn, blastp and blastx from the National Centre for Biotechnology Information (http://blast.ncbi.nlm.nih.gov/).

### Rapid amplification of cDNA ends (RACE) of the receptor for *D. pulex* RPCH (Dappu-RPCHR) and making of the construct for receptor screens

For the initial amplification of the RPCH receptor of *D. pulex*, primers were designed for use in 5′ and 3′ RACE PCRs with the Roche 5′/3′ RACE kit 2nd generation and RNA derived from animals in RNA-later.

For 3′ RACE PCR, cDNA was synthesised with the supplied oligo dT-anchor primer (Roche 8 adapter, R8, Roche). The cDNA was used in a nested PCR to amplify the 3′end in 50 µl reactions using MyTaq DNA polymerase (Bioline): (i) firstly, with the gene-specific forward primer 1 (gspF1) and the anchor PCR Primer (R9, Roche), and employing the following thermal cycling conditions for amplification: initial denaturation at 95 °C for 2 min, 35 cycles of 95 °C for 15 s, 52 °C annealing for 30 s and 72 °C for 10 s; followed by a final elongation step of 72 °C for 10 min. The resulting PCR was cleaned with the Wizard® SV Gel and PCR Clean-Up System (Promega Corporation), diluted 1:20 with water and used as template DNA in the second PCR with the same cycling conditions as described in (i) above, but with the gene-specific forward primer 2 (gspF2) and the gene-specific reverse primer 1 (gspR1).

For 5′ RACE PCR, cDNA was synthesised using the SuperScript™ III First Strand Synthesis System for RT-PCR (Invitrogen) with a gene-specific reverse primer 1 (gspR1); the resulting cDNA solution was cleaned with the Wizard® SV Gel and PCR Clean-Up System (Promega Corporation), the cDNA was eluted in nuclease-free water and the terminal transferase reaction was carried out as instructed (5′ RACE kit protocol, Roche). The resulting dA-tailed cDNA was then amplified by nested PCR as follows in 50 µl reactions using MyTaq DNA polymerase (Bioline): (i) with oligo-dT anchor primer R8 (Roche) and reverse primer 2 (gspR2), subjected to the following thermal cycling regime:- initial denaturation at 95 °C for 2 min, 10 cycles of 95 °C for 15 s, 52 °C for 30 s and 72 °C for 40 s; followed by 25 cycles of 95 °C for 15 s, 52 °C for 30 s and 72 °C for 40 s + 20 s/cycle, and a final elongation step of 72 °C for 10 min. (ii) 1 µl of the above reaction, R9 primer (Roche) and reverse primer 3 (gspR3), were cycled as follows: initial denaturation at 95 °C for 2 min, followed by 35 cycles of 95 °C for 15 s, 52 °C for 30 s and 72 °C for 40 s and a final elongation step of 72 °C for 10 min. PCRs were repeated with Expand Hi Fi taq polymerase (Roche).

Transmembrane regions of the amplified receptor were predicted by TM predictor in ExPASy Bioinformatics Resource Portal (Swiss Institute of Bioinformatics: www.expasy.org), while the signal peptide was predicted by SignalP 4.0^38^, accessed via http://www.cbs.dtu.dk/services/SignalP/.

For the cell screens, the full RPCHR sequence was amplified by a PCR reaction using a specific forward (5′-CACCATGTGTTCCAACGACAGCA-3′) primer with the ‘CACC’ Kozak sequence added to the 5′ side to facilitate translation in mammalian cells^39^ and reverse (5′-TTAAAATATATGTGTGACGACAGTTG-3′) primer. The PCR mixture was composed of 0.5 µl Advantage II polymerase mix (Clontech), 5 µl 10x Advantage PCR buffer (Clontech), 1 µl dNTP mixture (10 mM each), 1 µl forward and reverse primers (10 µM), 38.5 µl water and 3 µl cDNA. The amplification program consisted of an initial denaturation step of 95 °C for 180 s, followed by 35 cycles of 94 °C for 30 s, 60 °C for 60 s, 68 °C for 2 min and a final elongation step of 68 °C for 10 min. The analysed and purified PCR product was subsequently cloned into a pcDNA3.1/V5-His-TOPO TA expression vector (Invitrogen) and sequenced using T7 and BGH primers (LGC Genomics). Bacteria containing the plasmids with the insert of the correct sequence and right orientation were transferred into 150 ml LB medium with ampicillin (100 µg/ml, Invitrogen) and grown overnight at 37 °C in a shaking incubator. Subsequently the plasmid was isolated by means of EndoFree Plasmid Maxi Kit (Sigma-Aldrich) and once again the sequence was confirmed.

### Phylogenetic analyses

We compared the Dappu-RPCH sequence with other members of the AKH/ACP/Crz/GnRH family. The same was done for the receptor sequences. All analyses were performed with the MEGA software vers. 6^40^. We aligned the amino acid sequences of a selection of AKH and RPCH precursor genes from insects and crustaceans. In addition, we aligned the amino acid sequences of the Dappu-RPCHR with three pharmacologically characterised receptors of the silk moth, *Bombyx mori*, viz. Bommo-AKHR, -CrzR and -ACPR. Alignments were performed by using MUSCLE (Multiple Sequence Comparison by Log- Expectation). Identical and similar residues were calculated with BLASTP. In addition, a phylogenetic tree of a selection of receptors was constructed after alignment with MUSCLE with the neighbour-joining method (1000-fold bootstrap resampling) using the Jones Taylor Thornton mutation data matrix.

### Cell culture and transfections

Pharmacological analyses were performed in Chinese hamster ovary (CHO) WTA11 cells stably co-expressing the bioluminescent protein apoaequorin and the promiscuous G_α16_ subunit, which couples most agonist-induced GPCRs to the phospholipase C and Ca^2+^ pathway, irrespective of their natural signalling cascade^41^.

CHO-WTA11 cells were cultured in monolayers in Dulbecco’s Modified Eagles Medium nutrient mixture F12-Ham (DMEM/F12) with L-glutamine, 15 mM HEPES, sodium bicarbonate and phenol red (Sigma-Aldrich) supplemented with 100 IU/ml penicillin and 100 µg/ml streptomycin (Gibco), 10% heat-inactivated fetal bovine serum (Gibco) and 250 mg/ml zeocin (Gibco). All cells were maintained in an incubator at 37 °C with a constant supply of 5% CO_2_.

Transfections with pcDNA3.1-Dappu-RPCHR or empty pcDNA3.1 vector were carried out in T75 flasks at 60–80% confluency. Transfection medium was prepared using the Lipofectamine LTX Kit (Invitrogen) with 2.5 ml Opti-MEM® (Gibco), 12.5 µl PlusTM Reagent and 5 µg vector construct in 5 ml polystyrene round-bottom tubes. After a 5 min incubation period at room temperature, 30 µl LTX was added to the medium. After a further incubation period of 30 min at room temperature, the medium was removed and DNA/LTX mix was added dropwise to the cells followed by 3 ml of fresh complete medium.

Following transfection, cells were incubated overnight (37 °C, 5% CO_2_), then 10 ml of cell medium was added followed by a second overnight incubation (37 °C, 5% CO_2_). Ligand-induced changes in intracellular Ca^2+^ were then monitored in the cells as described below.

### *In vitro* bioluminescence assay

CHO-WTA11 cells transfected with receptor construct (or empty vector) plasmid were detached with PBS, complemented with 0.2% EDTA (pH 8.0), and rinsed off the flask with DMEM/F12 with L-glutamine and 15 mM HEPES (Sigma-Aldrich). The number of viable and nonviable cells was determined using a TC20 automated Cell Counter (BIO-RAD). The cells were pelleted for 5 minutes at 800 rpm and resuspended to a density of 5.10^6^ cells/ml in sterile filtered bovine serum albumin (BSA) medium (DMEM/F12 with L-glutamine and 15 mM Hepes, supplemented with 0.1% BSA) and loaded with 5 µM Coelenterazine_h (Invitrogen) for 4 h in the dark, at room temperature, while gently shaking to reconstitute the holo-enzyme aequorin. After a 10-fold dilution, cell solution (50 µl) was delivered to each well in a 96-well plate (~25000 cells/well) and cells were exposed to potential ligands reconstituted in BSA medium and distributed across the plate. In every row, BSA medium alone was placed in one well to serve as the negative control (blank). The Ca^2+^ response was recorded for 30 s on a Mithras LB 940 (Berthold Technologies). After 30 s, 0.1% Triton X-100, a non-ionic surfactant, was added to the same well to measure the total cellular Ca^2+^ response. The total response (ligand + Triton X-100), which is directly related to the number of cells present in the well, was used to normalise the response. The response of each negative control was subtracted from the luminescence obtained for wells within the same row. Calculations were made using the output file from the Microwin software (Berthold Technologies) in Excel (Microsoft). Further analysis was done in GraphPad Prism 5.

### Synthetic peptides

Peptides were custom-synthesized either by Dr Kevin D. Clark (Department of Entomology, University of Georgia, USA), or were purchased from Peninsula Laboratories (Belmont, CA, USA), from Pepmic Co., Ltd. (Suzhou, China), or Synpeptide Co. (Shanghai, China). For primary structures see Tables 1 and 2.

The peptides were dissolved in 80% acetonitrile and purified with reversed-phase high performance liquid chromatography (RP-HPLC). The bicinchoninic acid (BCA) method was applied to determine the concentrations of RP-HPLC-purified peptides^42^. The purity of RP-HPLC fractions was verified with a matrix-assisted laser desorption/ionization tandem time-of-flight (MALDI TOF/TOF, Ultraflex II Bruker Daltonics) mass spectrometer.

### Data Availability

All data generated or analysed during this study are included in this published article (and its Supplementary Information files).

## Electronic supplementary material

Supplementary Information
