# Supplementary material for: Characterisation and pharmacological analysis of a crustacean G protein-coupled receptor: the red pigment-concentrating hormone receptor of Daphnia pulex
Source: Sci Rep. 2017 Jul 31;7:6851. doi: 10.1038/s41598-017-06805-9 (PMC5537346; doi:10.1038/s41598-017-06805-9)
Supplement: Supplementary file 1 — Supplementary Information [file 41598_2017_6805_MOESM1_ESM.pdf]

Characterisation and pharmacological analysis of a crustacean G protein-coupled receptor: the red pigment-concentrating hormone receptor of *Daphnia pulex*

Heather G. Marco[1, a, *], Heleen Verlinden[2, a], Jozef Vanden Broeck[2] and Gerd Gäde[1]

[1]Department of Biological Sciences, University of Cape Town, Rondebosch, South Africa

[2] Molecular Developmental Physiology and Signal Transduction, KU Leuven, Naamsestraat 59, P.O. Box 02465, B-3000 Leuven, Belgium.

[a]Equally contributing first authors

*Corresponding author: Dr. HG Marco, Department of Biological Sciences, University of Cape Town, Rondebosch, 7700, South Africa. Email: Heather.Marco@uct.ac.za

Supplemental Table 1: Primer list used in this study for amplifying the open reading frame of *rpch* and *rpchr* in *Daphnia pulex*.

Open reading frame	Primer	Annealing temp. (^{o}C)
Dappu-RPCH	**Dpf**: 5'-GCT ATT GAT GAT CGG CCT GGC C-3'	52
	**Dpr**: 5'-TAC GGA TGT AAA GCC TAC ATG TCC T-3'	52
	**Dpr2**: 5'-CAT CCT GCC ATC TAG CGG TG-3'	52
	**Dpr3**: 5'-GAC CAA CAA GAG AGA TTT CAG-3'	55
	**Dpr4**: 5'-GGA TAG GCA AGG ACT TGG TAG AG-3'	55
Dappu-RPCHR	**gspF1**: 5'-ATG TCG ACA GTT CAG TTT GAC G-3'	52
	**gspF2**: 5'-ACG ATT TCC AAC GCC TGC GCT T-3'	52
	**gspR1**: 5'-TTA AAA TAT ATG TGT GAC GAC AGT TGG-3'	52
	**gspR2**: 5'-CGT CAG CAG TGG CAA CCG GTA G-3'	52
	**gspR3**: 5'-GAG AGC AAA TGA CGG AAC AAG C-3'	52

```
DAPPU RPCH SCAF    MANHRILILTLLMIGLASAQVNFSTSWGKRSPSTSTKAAEPPSAPSYRQNFHSKKVEPGTLETLPNNQHLPESFDTVSSTIYDDAEEQRISISLPSPCLSILKSLLLVNQIVEVK---
DAPPU RPCH EST                 MIGLASAQVNFSTSWGKRSPSTSTKAAEPPSAPSYRQNFHSKKVEPGTLETLPNNQHLPESFDTVSSTIYDDAEEQRISISLPSPCLSILKSLLLVNQIVEVK---
DAPPU RPCH GER                 MIGLASAQVNFSTSWGKRSPSTSTKAADPPSAPSYRQNFHSKKVEPGTLESLPNNQHLPESFDTVSSTIYDDAEEQRISISLPSPCLSLLKSLLLVNQIVEVK---
DAPPU RPCH NCBI  MDMANHRILILTLLMIGLASAQVNFSTSWGKRSPSTSTKAAEPPSAPSYRQNFHSKKVEPGTLETLPNNQHLPESFDTVSSTIYDDAEEQRISISLPSPCLSILKSLLLVNQIVEFKNSP
DAPPU RPCH PRED    MANHRILILTLLMIGLASAQVNFSTSWGKRSPSTSTKAAEPPSAPSYRQNFHSKKVEPGTLETLPNNQHLPESFDTVSSTIYDDAEEQRISISLPSPCLSILKSLLLVNQIVEFKNSP

DAPPU RPCH SCAF  -----------YLF-------------
DAPPU RPCH EST   -----------YLF-------------
DAPPU RPCH GER   -----------YLLKFKTN--------
DAPPU RPCH NCBI  LDGRMHRFKIENLFPLPNRTCRLYIRR
DAPPU RPCH PRED  LDGRMHRFKIENLFPLPNRTCRLYIRR
```

Supplemental Figure 1: An alignment of the predicted *Daphnia pulex* preprohormone RPCH sequences obtained from searching the genomic scaffolding sequence (SCAF) and the expressed sequence tag (EST) information available from www.wfleabase.org[7]; the predicted genomic scaffold sequence annotated and added to the NCBI database[9] (NCBI), the predicted sequence mined from the genome[8] (PRED), and the deduced sequence following cDNA amplification from a German *D. pulex* ecotype (GER; current study).

The black box represents amino acids that are 100% identical, the grey boxes represent amino acids that are similar in charge, cleavage sites are indicated by arrows (↓) and sequences that are underlined are predicted signal peptides. The alignment was performed in the bioinformatics tool BioEdit and gaps (-) have been introduced to optimise the alignment[37].

```
GCTATTG

Dpf  →
ATGATCGGCCTGGCTGGCGCTCAGGTGAATTTCTCGACCAGCTGGGGCAAGCGCTCACCT
 M  I  G  L  A  G  A  Q  V  N  F  S  T  S  W  G  K  R  S  P

TCGACTTCGACGAAAGCAGCGGATCCACCTTCAGCTCCATCTTACCGCCAAAATTTCCAT
 S  T  S  T  K  A  A  D  P  P  S  A  P  S  Y  R  Q  N  F  H

TCGAAAAAGGTCGAGCCAGGGACGCTGGAATCGCTCCCCAATAATCAGCATCTTCCCGAA
 S  K  K  V  E  P  G  T  L  E  S  L  P  N  N  Q  H  L  P  E

TCGTTCGACACCGTTTCCTCGACGATTTACGACGACGCCGAAGAGCAACGAATCAGCATA
 S  F  D  T  V  S  S  T  I  Y  D  D  A  E  E  Q  R  I  S  I
              ←  Dpr4                  ←  Dpr3
TCTCTACCAAGTCCTTGCCTATCCCTTCTGAAATCTCTCTTGTTGGTCAATCAAATAGTC
 S  L  P  S  P  C  L  S  L  L  K  S  L  L  L  V  N  Q  I  V

GAGGTAAAATATCTTTTAAAATTTAAAACAAATTAA
 E  V  K  Y  L  L  K  F  K  T  N  *
                                 ←  Dpr2
AATCTCAAAATAGTTAAAAAATTCACCGCTAGATGGCAGGATGCATAGGTTAAAATAGAA
                                    ←  Dpr
AATCTTTTCCCGTTGCCAATAGGACATGTAGGCTTTACATCCGTA
```

Supplemental Figure 2: The RPCH preprohormone cDNA and deduced amino acid sequence of D. pulex (German maternal clone). Underlined sequences represent the 5' and 3' untranslated region (UTR), the green box represents the signal peptide region, the grey box the pro-RPCH peptide sequence, the blue box the dibasic cleavage sites, the amino acid sequence in italics represents the "RPCH precursor-related peptide" (RPRP), the purple box the regions where the forward and reverse primer binds, the yellow box the region where primers for 5' RACE binds, and the letters in bold represent the start and stop codons.

```
AGAGTGTGAGGCTTATACCGTTCCGCCCGCACGCCGAACGACCGAGGCGCAGCGAGTCAGGGAGGCGAGGAAGCGAAGAGCGCCCAATAC
GCAACCGCCTCTCCCCGCCGCGTGCCGATTCATTAATGCAGCTGCACGACAGGTTTCCCGACTGAAAGCGGCAGTGAGCGCAACGCAATA
TGTGAGTAGCTCACTCATAGGCACCCAGGCTTTACACTTTATGCTCCGGCTCGTATGTGTGTGAATGTGAGCGGATACATTTCACACAGG
AAACAGCTATGACCATGATTACGCCAAGCTATTTAGGTGACACTATAGAATACTCAAGCTATGCATCCAACGCGTTGGGAGCTCTCCCAT

ATGGTCGACTGCAGGCGGCCGCGAATTCACTAGTGATTCCTCCAGTCTCAGTCAGCATTCGAACGTCCACTCTCGTCATTCAGCATTTAG
                                                                    gspF1 →            gspF2 →
CACAACTCTTTTGATTCCCAAGTGAAAACCGTGCGACAGCTGCACCATTCCCGTCCATCCAGTGGAATTTGACGATTTCCAACGCCTGCG

CTTTCAGAAGCCGTTACTCCGTTTCAGCGAA
                               ATGTGTTCCAACGACAGCAGCAGCCTCAGCGGCAATATGTGCATGATGACTGAACGGGA
                               M  C  S  N  D  S  S  S  L  S  G  N  M  C  M  M  T  E  R  D

TGCGTCGTTTAGCGGTGAGAGCACCGTCAACCCAGAGTTTGACAGTGGCAGTAGTATTAGTGGCGGAAGCAGCAGCACCACTGCGGTCGA
  A  S  F  S  G  E  S  T  V  N  P  E  F  D  S  G  S  S  I  S  G  G  S  S  S  T  T  A  V  D

CTTATCCATGTTGCCGATCGACATGACTTTCAACGACGGCCATATTGTGTCCATCGCCACCTACAGTGTCTTGCTTATCATCTCAGTCTG
  L  S  M  L  P  I  D  M  T  F  N  D  G  H  I  V  S  I  A  T  Y  S  V  L  L  I  I  S  V  C
                                                                                  TMI
CGGCAACATCACCGTCCTAGTAAATCTGATCAAAAGGCGACACATCAGCAACCCGCGTGTCAATATTATGCTGACTCATCTAGCCATCGC
  G  N  I  T  V  L  V  N  L  I  K  R  R  H  I  S  N  P  R  V  N  I  M  L  T  H  L  A  I  A
                                                                                     TMII
CGACCTGCTGGTTACGTTGCTATTGATGCCGATAGAGATTGGATGGGCCGCTACGGTCCAGTGGAGAGCAGGTGATTTTTCCTGCCGGAT
  D  L  L  V  T  L  L  L  M  P  I  E  I  G  W  A  A  T  V  Q  W  R  A  G  D  F  S  C  R  I

TCTGGCCTTTTTCCGGACGTTCGGCTTGTTCCTCTCTTCATTTGTTTTGGTTTGCATCAGCATTGATAGATTCGGAGCTATTTTGCAACC
  L  A  F  F  R  T  F  G  L  F  L  S  S  F  V  L  V  C  I  S  I  D  R  F  G  A  I  L  Q  P
                           TMIII                                   ← gspR3
CATGAAATTAGATTATTGGAAACGTCGTGGGCGTTTCATGTTAGCCATTGCTTGGGCTTGTTCCGTCATTTGCTCTCTGCCTCAGGTTTT
  M  K  L  D  Y  W  K  R  R  G  R  F  M  L  A  I  A  W  A  C  S  V  I  C  S  L  P  Q  V  F
                                                            TMIV
CGTGTTCCACGTCAAAGCTCATCCGGAATACCCTTGGTATGAGCAGTGCGTCACTTTTGATTCGTTTCCGACCAAGGCTCACGAGATTTC
  V  F  H  V  K  A  H  P  E  Y  P  W  Y  E  Q  C  V  T  F  D  S  F  P  T  K  A  H  E  I  S

TTACGCTGCCTTCGGCATGATGATGATGTATGTTCTTCCACTCGCCGTTTTCGTCTTCACTTATAGTTCGATTCTTTGCGAGATCAGCAG
  Y  A  A  F  G  M  M  M  M  Y  V  L  P  L  A  V  F  V  F  T  Y  S  S  I  L  C  E  I  S  R
                                    TMV
GAGGAGCAAGGAAGTCGGACAAGAGGAGGGTATCCGAAGGGTGACTGTCGGTACGCTTGGACGAGCACGTATAAAAACCGTCAAAATGAC
  R  S  K  E  V  G  Q  E  E  G  I  R  R  V  T  V  G  T  L  G  R  A  R  I  K  T  V  K  M  T

TCTAGTGATCATTTCCGTTTTCATTTTCTGCTGGACGCCGTACAACATCATGAGCATCTGGTTCTGGTGTGACAGAGATTCGGCGCTTCA
  L  V  I  I  S  V  F  I  F  C  W  T  P  Y  N  I  M  S  I  W  F  W  C  D  R  D  S  A  L  Q
                                    TMVI
AGTTGACCAACGAATCCAAAAAGGCCTTTTTCTTTTTGCGTGTACCAATTCTTGCTTCAACCCTATGGTGTACGGTTACTTTTCGCGACG
  V  D  Q  R  I  Q  K  G  L  F  L  F  A  C  T  N  S  C  F  N  P  M  V  Y  G  Y  F  S  R  R
                                                            TMVII
AACGGTTCGCAGATCTCACCACGAACTCCACAGAAAGGTGGTCTATCATCCCAGTCGAATGGTATCAAGGGCCTTGGGTCCCTCACTCAG
  T  V  R  R  S  H  H  E  L  H  R  K  V  V  Y  H  P  S  R  M  V  S  R  A  L  G  P  S  L  R
                                                                               ← gspR2
AGTAGATTGCAATTCTAAATCATTGGAAGGAGCAGTAACTGAACCGTGTTTGCCACAGCAGCATCTACCGGTTGCGACTGCCGACGATCC
  V  D  C  N  S  K  S  L  E  G  A  V  T  E  P  C  L  P  Q  Q  H  L  P  V  A  T  A  D  D  P

TTCATGTCAGTTGGGACAGGCAGAACCGATTTTCACCGCCTCCTCTATCGCCGTAGCTGTTAAAAGAAGTAATAGCTGGCTGCTGAATCA
  S  C  Q  L  G  Q  A  E  P  I  F  T  A  S  S  I  A  V  A  V  K  R  S  N  S  W  L  L  N  H
                            ← gspR1
TCGATCGAATCCAACTGTCGTCACACATATATTTTAA
 R  S  N  P  T  V  V  T  H  I  F  *
```

Supplemental Figure 3: The RPCH Receptor cDNA and its deduced peptide sequence amplified in Cape Town from a German *D. pulex* maternal clone. Underlined sequences represent the 5' and 3' UTR, the black and grey boxes represent the transmembrane α-helices (TM I- VII), primers used in 5'/ 3' RACE are in purple boxes with arrows indicating their direction and letters in bold represent the start and stop codons. Motifs that are known to be characteristic of rhodopsin-like GPCRs are shown in bold letters in the TM regions: helix 1:- GN, helix 2:- HLX_3DX_8P, helix 3:- $SX_3LX_2IX_2DR$, helix 4:-WX_8P, helix 5:- PX_7Y, helix 6:- FX_2CWXP, and helix 7:- $LX_4NSX_2NPX_2Y$.

```
GNO_748024 MSTVQFDDFQRLRFQKPLLRFSEMCSNDSSSLSGNMCMMTERDASFSGESTVNPVFDSASSISGGSSSSTTTTVDLSMLP
ACD75498
KY426816                          MCSNDSSSLSGNMCMMTERDASFSGESTVNPEFDSGSSISGGSSS--TTAVDLSMLP

GNO_748024 IDMTFNDGHVVSIATYSVLLIISVCGNITVLVNLIKRRHISNPRVNIMLTHLAIADLLVTLLLMPIEIGWAATVQWRAGD
ACD75498              SIATYSVLLIISVCGNITVLVNLIKRRHISNPRVNIMLTHLAIADLLVTLLLMPIEIGWAATVQWRAGD
KY426816   IDMTFNDGHIVSIATYSVLLIISVCGNITVLVNLIKRRHISNPRVNIMLTHLAIADLLVTLLLMPIEIGWAATVQWRAGD
                      *********************************************************************

GNO_748024 FSCRMLAFFRTFGLFLSSFVLVCISIDRFGAILQPMKLDYWKRRGRFMLAIAWACSVICSLPQVFVFHVKAHPEYPWYEQ
ACD75498   FSCRMLAFFRTFGLFLSSFVLVCISIDRFGAILQPMKLDYWKRRGRFMLAIAWACSVICSLPQVFVFHVKAHPEYPWYEQ
KY426816   FSCRILAFFRTFGLFLSSFVLVCISIDRFGAILQPMKLDYWKRRGRFMLAIAWACSVICSLPQVFVFHVKAHPEYPWYEQ
           ****:***************************************************************************

GNO_748024 CVTFDSFPTKAHEISYAAFGMMMMYVLPLAVFVFTYSSILCEISRRSKEE----EGIRRVTVGTLGRARIKTVKMTLVII
ACD75498   CVTFDSFPTKAHEISYAAFGMMMVYVLPLAVFVFTYSSILCEISRRSKEAVGQEEGIRRVTVGTLGRARIKTVKMTLVII
KY426816   CVTFDSFPTKAHEISYAAFGMMMMYVLPLAVFVFTYSSILCEISRRSKE-VGQEEGIRRVTVGTLGRARIKTVKMTLVII
           ***********************:*************************:::::**************************

GNO_748024 SVFIFCWTPYNIMSIWFWCDRDSALQVDQRIQKGLFLFACTNSCFNPMVYGYFSRRTVRRSHHELHRKVVYHPSRMASRA
ACD75498   SVFIFCWTPYNIMSIWFWCDRDSALQVDQRIQKGLFLFACTNSCFNPMVYGYFSRRTVRRSHHELHRKVVYHPSRMASRA
KY426816   SVFIFCWTPYNIMSIWFWCDRDSALQVDQRIQKGLFLFACTNSCFNPMVYGYFSRRTVRRSHHELHRKVVYHPSRMASRA
           ********************************************************************************

GNO_748024 LGPSLRVDCNSKSLEGAATEPCLPQQHLPVATADGPSCQ-GQAEPIFTASSIAVAVKRREHL
ACD75498   LGPSLRVDCNSKSLEGAATEPCLPQQHLPVATADGPSCQ-GQAEPIFTASSIAVAVKRSNSWL-NNRSNPTVVTHIF
KY426816   LGPSLRVDCNSKSLEGAVTEPCLPQQHLPVATADDPSCQLGQAEPIFTASSIAVAVKRSNSWLLNHRSNPTVVTHIF
           *****************:****************:****:******************
```

Supplemental Figure 4: A comparison of the Dappu-RPCH receptor sequence amplified in the current study (GenBank acc. no. **KY426816**) that was derived from a German *Daphnia pulex*, the genomic sequence (**GNO_748024**) and an EST sequence (GenBank acc. no. **ACD75498**). Differences of the predicted sequences with our functional sequence are shaded.

```
SagveRPCHR
DappuRPCHR MCSNDSSSLSGNMCMMTERDASFSGESTVNPEFDSGSSISGGSSS-------TTAVDLSM  53
BommoAKHR                                MDIDEKVSGPGGASQKNWSHLLHVNNTYDE  30

                                   TM1 •                         •   •
SagveRPCHR                                        RNRHRKSRVNMMIMHLSLADM  20
DappuRPCHR LPIDMTFNDGHIVSIATYSVLLIISVCGNITVLVNLIKRRHI-SNPRVNIMLTHLAIADL 112
BommoAKHR  LPLEMRFNYSHMVSMTVYSVLMVISATGNLTVLYQLVRRRRAKRASRLDILLMHLAVADL  90

            TM2        •            •          •TM3         ••      *
SagveRPCHR IVTFVFLPTEITWHITIEWVFGNFGCKIYKFFSAFGFYMSSMVLVCISLDRYFAVLHPLK  80
DappuRPCHR LVTLLLMPIEIGWAATVQWRAGDFSCRILAFFRTFGLFLSSFVLVCISIDRFGAILQPMK 172
BommoAKHR  MVTFLMMPLEIAWAGTVQWFAGDLMCRVMMFTRTFGLYLSSFVLICIAVDRYYAILKPLN 150

                             TM4   •*   *            *•
SagveRPCHR VNDAQRRGKIMLFFAWLISAVISLPQSVIFNVQPHPKYADFHQCVTFGFFNSNNGGEMMY 140
DappuRPCHR LDYWKRRGRFMLAIAWACSVICSLPQVFVFHVKAHPEYPWYEQCVTFDSFPTK-AHEISY 231
BommoAKHR  VT-WEATVRRAIIVAWVCAGLASLPQSFIFHVEEHPEVKGYNQCVSYGSLPTE-KHEFAY 208

                  TM5 •*                                              *
SagveRPCHR TIFCISFLYFIPLSIIIIAYSRIILEISRKSRDTQHDYSREERYHGRLQLRRSNMSNIER 200
DappuRPCHR AAFGMMMMYVLPLAVFVFTYSSILCEISRRSKEVGQ----------EEGIRRVTVGTLGR 281
BommoAKHR  FLVNMILMYVIPLVSTLYCSCAALFEIIRRANT--A----------NDKMRRSGIGLLGR 256

                      TM6 •     •                                 TM7 •
SagveRPCHR ARTRTLRMTFIIVMAFIWCWTPYALATLWNFIDPVTFYSMNKHVQDILFIIAVSNSVVNP 260
DappuRPCHR ARIKTVKMTLVIISVFIFCWTPYNIMSIWFWCDRDSALQVDQRIQKGLFLFACTNSCFNP 341
BommoAKHR  ARARTLKMTVTIVLVFFTCWSPYYCYCLWYWIDKESIKNLDPALQKAMWLFSCTNSCANP 316

             •*
SagveRPCHR IIYGRYSISCCRGLWNKAGDCCCWCCCWC                                289
DappuRPCHR MVYGYFSRRTVRRSHHELHRKVVYHPSRMASRALGPSLRVDCNSKSLEGAVTEPCLPQQH 401
BommoAKHR  IVYGVFNRNRWNWRAGKFQNGRCR----SGSGRKGSRLPH--------GESTEI--SAAT 362

SagveRPCHR
DappuRPCHR LPVATADDPSCQLGQAEPIFTASSIAVAVKRSNSWLL---NHRSNPTVVTHIF        451
BommoAKHR  LSRARHSNGSDHNGRRDSSYA-----NQNGPQKHWNTINNNHVTNGMV             405
```

Supplemental Figure 5: A comparison of the Dappu-RPCH receptor sequence with the predicted RPCH receptor sequence of the spiny lobster *Sagmariasus verreauxi* and the *Bombyx mori* AKH receptor sequence. Identical residues between the Dappu-RPCHR and any of the other receptors are highlighted in black, and conservatively substituted residues in grey. Dashes indicate gaps that were introduced to maximise homologies. Putative transmembrane regions (TM1–TM7) are indicated by grey bars. Conserved amino acids for the rhodopsin-like GPCRs are indicated by a dot (•), whereas conserved amino acids for the GnRH receptor subfamily are indicated by asterisk.
